# Conditional Knockout of Cav2.1 Disrupts the Accuracy of Spatial Recognition of CA1 Place Cells and Spatial/Contextual Recognition Behavior

**DOI:** 10.3389/fnbeh.2016.00214

**Published:** 2016-11-03

**Authors:** Dahee Jung, Yu J. Hwang, Hoon Ryu, Masanobu Kano, Kenji Sakimura, Jeiwon Cho

**Affiliations:** ^1^Center for Neuroscience, Korea Institute of Science and Technology Seoul, South Korea; ^2^Neuroscience Program, Korea University of Science and Technology Daejeon, South Korea; ^3^Center for Neuromedicine, Brain Science Institute, Korea Institute of Science and Technology Seoul, South Korea; ^4^VA Boston Healthcare System, Department of Neurology and Boston University Alzheimer’s Disease Centre, Boston University School of Medicine, Boston MA, USA; ^5^Department of Neurophysiology, Graduate School of Medicine, University of Tokyo Tokyo, Japan; ^6^Department of Cellular Neurobiology, Brain Research Institute, Niigata University Niigata, Japan

**Keywords:** P/Q type calcium channels, burst, hippocampus, place cell, learning and memory

## Abstract

Hippocampal pyramidal neurons play an essential role in processing spatial information as implicated with its place-dependent firing. Although, previous slice physiology studies have reported that voltage gated calcium channels contribute to spike shapes and corresponding firing rate in the hippocampus, the roles of P/Q type calcium channels (Cav2.1) underlying neural activity in behaving mice have not been well-investigated. To determine physiological and behavioral roles of Cav2.1, we conducted place cell recordings in CA1 and hippocampus dependent learning/memory tasks using mice lacking Cav2.1 in hippocampal pyramidal neurons under CamK2α-Cre recombinase expression. Results suggested that impairments shown in behavioral tasks requiring spatial and contextual information processing were statistically significant while general neurological behaviors did not differ between groups. In particular, deficits were more profound in recognition than in acquisition. Furthermore, place cell recordings also revealed that the ability to recollect spatial representation on re-visit in the conditional knockout was also altered in terms of the cue recognition while the capability of a place cell to encode a place was intact compared to the control group. Interestingly, CA1 pyramidal neurons of conditional knockout mice showed reduced burst frequency as well as abnormal temporal patterns of burst spiking. These results provide potential evidence that Cav2.1 in hippocampal pyramidal cells modulates temporal integration of bursts, which, in turn, might influence the recognition of place field and consequently disrupt spatial recognition ability.

## Introduction

Hippocampal pyramidal neurons exhibit location-dependent discharges, providing physiological evidence that the hippocampus is critical for spatial learning and memory (O’Keefe and Dostrovsky, 1971; O’Keefe, 1979). Place cells are known to display two distinct firing modes: single spikes or high frequency spike clusters, also known as bursts (Huxter et al., 2003). Previous studies have attempted to reveal molecular and electrophysiological mechanisms modulating burst firing and its roles in various levels. For example, at the synaptic level, burst firing is functionally implicated in more successful information processing due to its stronger summation of excitatory post-synaptic potentials (EPSPs) and consequent reliable neurotransmitter release compared to a single spike (Xu et al., 2012). In addition, burst firing is more effective in inducing synaptic plasticity and long term potentiation, facilitating highly reliable communication between neurons (Lisman, 1997; Izhikevich et al., 2003). In behaving mice, burst spiking of hippocampal place cells is more spatially tuned when forming place fields compared to single spiking and also relevant to hippocampal theta rhythm, as it takes up a high proportion of firing during 6–7 Hz oscillation (Harris et al., 2001). Interestingly, various states of bursts and theta rhythm have been correlated with neurological diseases and specific behavioral states such as goal identification, resting, and sleep (Harris et al., 2001; Ovsepian and Friel, 2008; Grienberger et al., 2014). Despite several suggestions aforementioned about the functional roles of burst spiking on information processing and behavioral effects, its specific roles on spatial representation of hippocampal place cells and modulatory function on behavior have not been revealed yet.

Calcium influx via voltage gated calcium channels (VGCCs), among other ion channels, influences neuronal excitability either directly by shaping discharges or indirectly through gene expression and neurotransmitter release (White et al., 2008; Gamelli et al., 2011) to consequently affect behavior. In particular, VGCCs are known to be one of the main modulators of intrinsic burst firing in the hippocampus. For instance, L-type calcium channels and R-type calcium channels are implicated in the modulation of burst firing in the hippocampus as well as hippocampal-dependent learning and memory (Metz et al., 2005; White et al., 2008; Gamelli et al., 2011). However, the role of P/Q type calcium channels (Cav2.1) in place cells has not been well-studied despite its high expression in the forebrain (Hillman et al., 1991). Previous studies *in vitro* have demonstrated that Cav2.1 plays a significant role in various cellular mechanisms such as generation of dendritic burst, properties of firing shape, and modulation of synaptic plasticity (Llinas et al., 2007; Liu and Friel, 2008). In particular, Cav2.1 expressed in post-synaptic regions has been reported to be involved in synaptic competition and elimination, in which a single synapse is selectively strengthened through modulating translocation of dendrites (Hashimoto et al., 2011). Also, Cav2.1 is a predominant source of Ca^2+^ influx for exocytosis of neurotransmitters in presynaptic regions via asynchronous release, and it is involved in facilitating/decreasing synaptic strength via short-term synaptic plasticity in response to neuronal firing frequency (Catterall and Few, 2008; Catterall et al., 2013). Recent studies have investigated the mechanism of Cav2.1 on synaptic plasticity in the hippocampus as well as the cerebellum, and its effects on hippocampal dependent behaviors have been implicated (Nanou et al., 2016). However, global Cav2.1 knockout mice demonstrate high rates of mortality due to ataxia and unstable respiration, which has greatly limited investigating the function of Cav2.1 in cognitive behaviors (Jun et al., 1999; Koch et al., 2013). Although, a recent study circumvented the lethal phenotype by using the Cre-loxP system under the control of the NEX promoter to delete Cav2.1 in the neocortex, the mice still displayed substantial emotional impairments including anxiety and seizure. These affective changes observed in this transgenic mice line may have interfered with their performance in learning and memory tasks, therefore, results could be inconclusive in its attempt to identify the role of Cav2.1 in spatial learning and memory and hippocampal place cell activity (Mallmann et al., 2013).

In the present study, we genetically ablated Cav2.1 mainly in hippocampal pyramidal cells using the CamK2α-Cre line, which could prevent affective disorders as reported in earlier studies using the same Cre line (Barbarese et al., 2013; Brigman et al., 2013), to investigate the role of the pyramidal Cav2.1 in spatial representation – a hippocampal-dependent cognitive behaviors – and spiking patterns of CA1 place cells.

## Materials and Methods

### Ethics Statement

All animal experiments were carried out in accordance with the guidelines set and approved by the Institutional Animal Care and Use Committee (IACUC) of Korea Institute of Science and Technology (Approval Number: 2015019).

### Animal

Male mice (C57BL/6) lacking Cav2.1 (P/Q type calcium channel) in the hippocampus were used as a conditional knockout group (Cav2.1 cKO) and their floxed Cav2.1 littermates were used as a control group. To obtain the designed mouse line, CamK2α -Cre donor line mice (Tsien et al., 1996), B6.Cg-TG T29-1Stl/J (Stock#005359) from The Jackson Laboratory (Bar Harbor, MA, USA), were mated with Cav2.1 floxed line (Hashimoto et al., 2011) to conditionally ablate Cav2.1 under the control of Cre recombinase-expression, which is restricted to mostly in CA1 regions and some in the forebrain. Cav2.1 exclusively on pyramidal neurons in the hippocampus and the cortex, therefore, were eliminated in Cav2.1 cKO mice (Cav2.1^lox/lox^ and CamK2α^+/Cre^) but not in control mice (Cav2.1^lox/lox^ and CamK2α^+/+^). All mice were genotyped using PCR before and after the experiments.

All mice were kept in home cages with free access to food and water in an alternating 12-h light–dark cycle. Mice at the age of >8 weeks were used for all experiments including histological, electrophysiological, and behavioral experiments. Different sets of mice were submitted to open field task and novel object task. One set of mice underwent behavioral experiments of Y-maze, Morris water maze, and fear conditioning task with rest interval of 5 days and 2 weeks, respectively, to minimize the interactions from the previous tasks.

### Histology

Immunofluorescence staining and confocal microscopy were used to determine expression of Cav2.1 (Alomone Labs, Ltd, Jerusalem, Israel) (1: 100) and NeuN (Millipore, Billerica, MA, USA) (1:500). The staining procedures were performed as previously described (Ryu et al., 2006). The specimens were incubated for 60 min with AlexaFluor 594 goat anti-rabbit (abcam, Cambridge, UK) (1:400) and alexafluor 488 goat anti-mouse (abcam, Cambridge, UK) (1:200) after incubation of the primary antibody. Images were analyzed using an A1 Nikon confocal laser scanning microscope (Nikon, Tokyo, Japan). In order to investigate the extent of reduction of Cav2.1 over hippocampal subregions and some neocortex regions, we calculate the intensity (%) of Cav2.1 expression in cKO compared to the control. *t*-test was performed to compare the group difference.

### Open Field

A white Plexiglass test box (40 cm × 40 cm × 40 cm) was used to measure spontaneous locomotor activity in an open field experiment as described in a previous study (Koh et al., 2008). Each mouse (control *n* = 7; Cav2.1 cKO *n* = 6) were habituated in the behavioral room for 30 min prior to the experiment to acclimate to the white noise and lighting. For the experiment, each mouse was placed in the center facing a wall and its activity was monitored for 30 min via Ethovision 3.1 (Noldus Information Technology, Leesburg, VA, USA). Total walking distance and percentage of time spent at the center of the chamber were calculated to assess locomotion and anxiety.

### Y-Maze

Y-maze test was performed in a Y-shaped Plexiglass with three identical arms (36 cm long, 12 cm high, and 3 cm wide floor becoming wider up to 10 cm wide at the top) as described in a previous study (Kim et al., 2013). Once placed in one of the arms of the Y-shaped maze, mice (control *n* = 9; cKO *n* = 9) were allowed to move freely through the arms for 8 min, during which all activity was videotaped. An entry was defined as a trial in which all four paws went in and out from one arm. Sequences of arm entries were analyzed, and success alternation was defined as consecutive entries of different arms in three trials. Alternation success was represented as following:

$$Alternation success(\%)=\frac{\#\mathrm{of}\;Success in Alternation}{\#of total entries - 2}\times 100.$$

### Novel Object Recognition

The testing apparatus for a novel object recognition experiment included a white Plexiglass (40 cm × 40 cm × 40 cm) and two objects, in unique shape and color, made of the same material to prevent any unintended preference over each other, as described in a previous study (Dix and Aggleton, 1999; Broadbent et al., 2010). Each mouse (control *n* = 7; cKO *n* = 9) was habituated to the arena for 30 min 1 day before the training. On the training phase, the mouse was placed in the box with two identical objects for 20 min. During the test phase on the following day, the mouse was re-exposed to the box for 10 min with one of the two objects switched to a different object. The object placed repeatedly from the training phase is referred to as ‘familiar objects,’ while the switched object in the testing phase is referred as ‘novel objects.’ All experiments were videotaped and scored by an experimenter blind to the genotypes. An observer counted the amount of time the mice spent exploring the objects. Exploration was defined as a behavioral epoch only when the animal headed directly toward the object within a distance of <2 cm. Time spent exploring each object during the test phase were then summed and compared. Discrimination index (DI) was calculated to measure the preference to each object, using the following equation:

$$\mathrm{Discri}\;\min\;ation Index(\mathrm{DI})=\frac{Time spent in new object-Time spent in old object}{Time spent in new object+Time spent in old object}.$$

### Morris Water Maze

Water maze with a hidden platform task was conducted as described in a previous study (D’Hooge and De Deyn, 2001). A circular water maze (diameter 1.2 m) was filled with opaque water (24°C) surrounded by a curtain with three cues attached. Mice (control *n* = 9; cKO *n* = 9) were randomly released from four starting points and forced to escape to a hidden platform (diameter 10 cm). Mice that failed to escape within 60 s were guided to the platform where they remained for 30 s (Park et al., 2015).

Each mouse underwent two consecutive trials twice per day with 1 h rest between the two trial blocks, for a total of four trials per day, for 7 days. Twenty four hours after the last training, the platform was removed from the pool, allowing the mouse to swim for 60 s for a probe test. All trials were video tracked via Ethovision 3.1 (Noldus Information Technology, Leesburg, VA, USA) for further analyses of swimming path.

### Contextual Fear Conditioning

Contextual fear conditioning test was conducted as described in a previous study (Rudy et al., 2004). The fear conditioning chamber with a stainless-steel floor was placed in a sound-proof box with a camera mounted on its ceiling (Med associates, Inc., St. Albans, VT, USA). On the first day of training, mice (control *n* = 9; cKO *n* = 9) were allowed to explore the chamber freely for 3 min, and received three foot shocks separated by 1 min (0.5 mA, 2 s). Twenty-four hours after the training, the mice were placed in the chamber for 10 min and their behavioral responses were videotaped. Freezing response, defined as an absence of any movement except breathing for >1 s, was scored twice by an experimenter that was blind to the genotypes then averaged.

### Extracellular Single Unit Recordings in Freely Moving Mouse

Under Zoletil anesthesia (30 mg/kg), mice were chronically implanted with a movable microdrive that consists of four tetrodes. Tetrodes were made up of four nichrome wires (Kanthal Precision Technology, Sweden), and the tip of each wire was gold-plated to obtain an optimal impedance level of 0.2–0.5 MΩ measured at 1 kHz. To record neuronal activity in the CA1 region, the tips of tetrodes were placed at coordinates of 1.4 mm lateral and 1.7 mm ventral to bregma, then the microdrive was secured onto the skull with dental cement. After 1 week recovery, tetrodes were lowered gradually until they reached the pyramidal layer of the CA1 region. To obtain unit signals, neural activity was sampled at 30,303 Hz via the Cheetah Data Acquisition System (Neuralynx, Tuscon, AZ, USA), amplified with gains of 5,000–20,000, and filtered at 600 Hz to 6 kHz. Upon successful identification of unit signals via unit screening process, a recording session was initiated in a recording chamber.

The recording chamber was made of a black acryl cylinder (diameter = 30 cm; high = 35 cm) with a white cue attached inside as a visual cue covering 90° arc. The recording chamber was enclosed by a black curtain to keep other objects from acting as an unintended cue. There were two recording sessions, separated by a 30 min interval. Environmental setup of two recording sessions was completely identical to measure spatial recognition ability of place cells under re-exposure.

Upon completing the recording sessions, mice were anesthetized with 10% Avertin, and currents (10–30 μA, 10 s) were passed through the electrode tip to verify its location. Afterward, the mice were perfused with 10% formalin solution diluted in 0.9% saline for brain extraction and the brains were preserved in 10% formalin solution for a day. Fifty μm-thick sections of a brain were stained with Cresyl Violet (Sigma, USA). Finally, the recording sites were determined by examining the marking lesion using a light microscope.

### Data Analysis

Spike data was isolated into single units using SpikeSort3D program (Neuralynx, USA). Only unit data that met place cell criteria (mean firing rate >0.2 Hz, spatial information >1.0 s bits/s, presence of burst spikes, refractory period >1 ms) (Skaggs et al., 1993; Kim et al., 2007) during both sessions were analyzed. For the place firing rate analysis, we obtained position data in pixel by monitoring the LED lights that were placed on the mouse’s head. Pixels that were visited for less than 1 s were excluded from the analysis. The number of spikes that fired in a pixel was divided by the time spent in that pixel to calculate firing rate maps. The place field size was defined as the area of pixels within which the firing rate exceeded the mean firing rate for each session. Other parameters, such as spatial information, selectivity, and coherence, were calculated as previously described (Cho et al., 2012; Park et al., 2015). For comparison of the parameters’ mean between groups, unpaired two-tailed *t*-test was calculated.

To compare place fields between two sessions, we used similarity as an indicator of resemblance of two place fields by calculating pixel-by-pixel correlations of firing rate from the two recording sessions and correlation values were transformed into Fisher’s *Z* score. In addition, we calculated max similarity by rotating the place map of the second session by 5° until we identified the ‘rotation angle’ that yielded the highest pixel-by-pixel correlation. To compare the difference between similarity and max similarity values, the difference was normalized to the similarity value. Then, the rotation angle for the max similarity value within 0 ± 45° was used to classify cells into ‘stay’ in proportion and ‘remapped’ for other than 0 ± 45° for cue spatiality index.

In assessing firing mode, we define a burst as a spike cluster consisting of at least two consecutive spikes with subsequent decreases in the amplitude within an interval of <15 ms (Muller and Kubie, 1987). Intervals of spike within a burst (IntraBI) were numbered as the first IntraBI (interval between the first and second spike within a burst), the second IntraBI (interval between the second and third spike within a burst), and so on. The joint probability density analysis was used to describe the temporal relationship between two consecutive IntraBIs by calculating the probability of the first IntraBI followed by the second IntraBI.

## Results

### Conditional Deletion of Cav2.1

**FIGURE 1:**
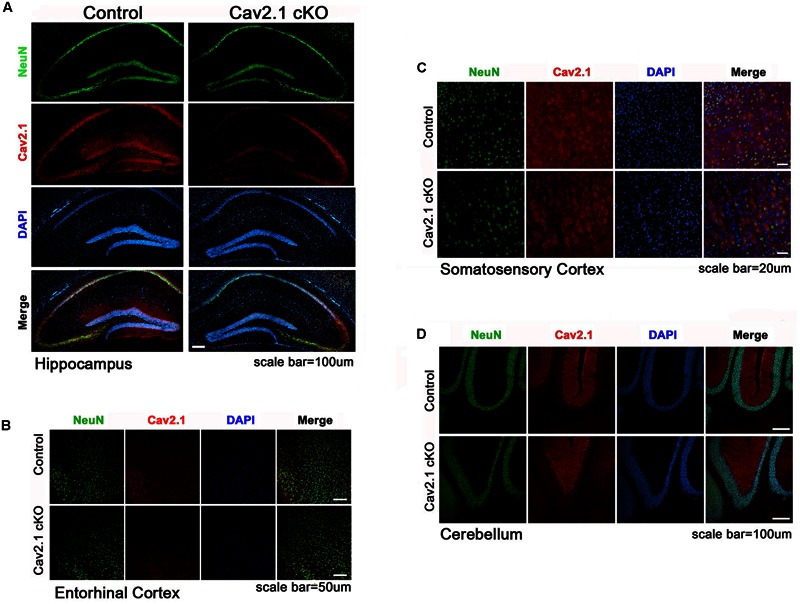
**Altered expression of Cav2.1 in Cav2.1 in immunohistology. (A)** Expression of Cav2.1 in the hippocampal CA1 was reduced in Cav2.1 cKO mice compared to control while neuronal viability was intact. **(B)** Expression of Cav2.1 channels was also reduced in the entorhinal cortex. **(C)** Expression of Cav2.1 in Sensorimoto cortex is reduced. **(D)** Both expression of Cav2.1 and viability of neurons in cerebellum were intact.

Transgenic mice carrying two loxP sequences flank inserted in exon 4 of Cav2.1 were used for Cre/loxP system as described previously (Hashimoto et al., 2011). To conditionally knockout Cav2.1 in pyramidal neurons in the hippocampus, the floxed Cav2.1 mice were crossed to CamK2α-Cre donor line, which is known to be expressed in mainly CA1 region starting at postnatal 21 day and spread to the neocortex in 2 month where αCamK2 is present. Quantifying the intensity of Cav2.1 immunoreaction in relative to the control group was conducted to show the extent of reduction across the subregions (**Figure 1**; Supplementary Figure S1). We confirmed that the level of Cav2.1 expression was decreased substantially in the hippocampus (expression level: 17.8% in CA1 *P* < 0.001, 25.5% in Dendate gyrus *P* < 0.001) and sensory/motor cortex (35.9%, *P* < 0.001), and also moderately in the entorhinal cortex (56%; *P* < 0.001) but not in hippocampal CA3 regions (115%; *P* = 0.26) of Cav2.1 cKO mice relatively to control mice. Also, the expression of Cav2.1 in the cerebellum, where Cav2.1 is known to be highly expressed, was completely intact (112%; *P* = 0.29). The histological data showed that deletion of Cav2.1 mainly occurred in the hippocampus, especially CA1 and the dendate gyrus regions, and moderately in the neocortex under the Cre-loxP system, whose effects over various neocortical regions were comparable with the results from previous studies (Barbarese et al., 2013; Brigman et al., 2013).

### Cav2.1 cKO Does Not Show Deficits in General Neurological Behaviors

**FIGURE 2:**
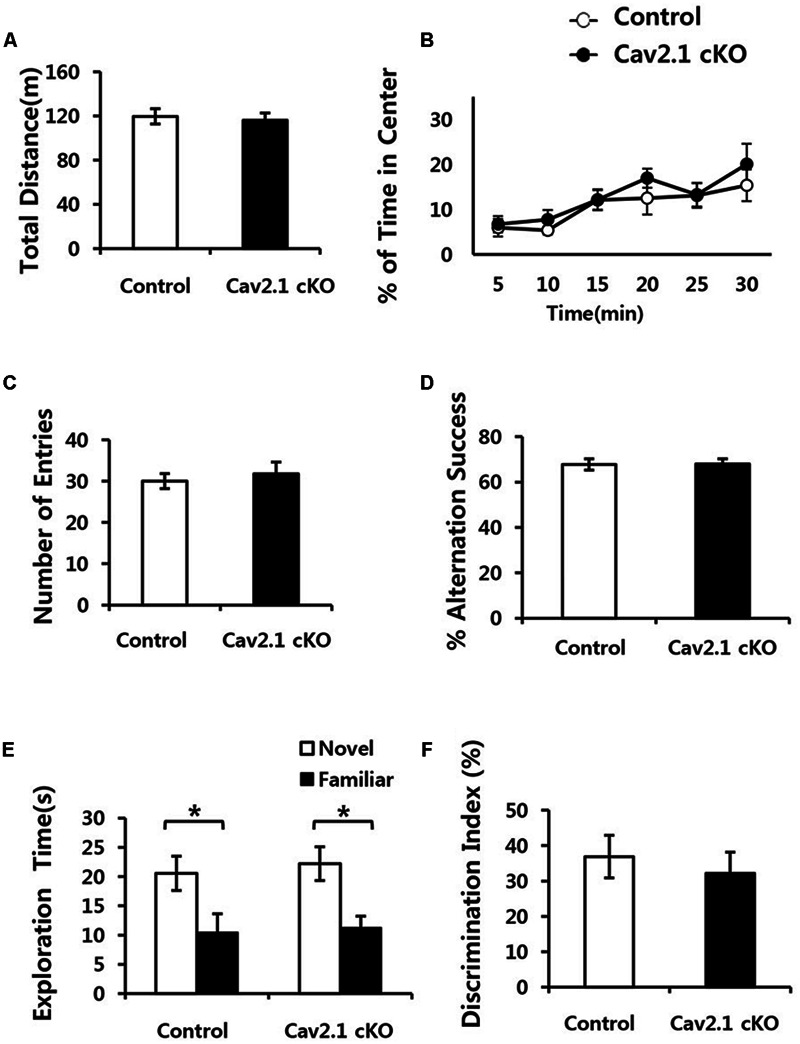
**Conditional deletion of Cav2.1 did not alter general neurological behaviors. (A,B)** Open field test. **(A)** Total distance traveled (cm) in both groups was not significantly different. **(B)** The % of time spent in the center area did not differ between groups (control, *n* = 7; cKO, *n* = 6). **(C,D)** Spontaneous alternation test. **(C)** The number of entries into the arms of Y-maze was not significantly different. **(D)** The % of alternation success in both groups were not significantly different (control, *n* = 9; cKO, *n* = 9). **(E,F)** Novel object recognition test. **(E)** Total exploration time and trends of exploration toward the familiar and novel objects were similar in both groups. **(F)** Discrimination indices were similar in both group (control, *n* = 7; cKO, *n* = 9). ^∗^*P* < 0.05.

To eliminate the possibility that general neurological deficits rather than changes in hippocampal-dependent behavioral ability in Cav2.1 cKO mice may contribute to altered performance on spatial and contextual memory task, we performed several general behavioral tasks (**Figure 2**).

First, locomotion and anxiety-like behavior were assessed through an open field task. As shown in **Figures 2A,B**, locomotor activity was normal in Cav2.1 cKO mice in that total locomotion distance during 30 min was not significantly different from control mice (control 119.7 ± 6.8 m; cKO 116.1 ± 6.7 m; *P* = 0.71). The percentage of time spent in the center of the field was measured as an indicator of anxiety-like behavior because rodents instinctively prefer to stay in peripheral areas when first introduced to a novel environment and gradually increase the tendency to explore the central area as time elapses. The ratio of time spent in the center gradually increased in both groups (significant effect of time *F*_(2.9)_ = 6.328, *P* = 0.002; no significant effect of group × trial interaction *F*_(2.9)_ = 0.341; *P* = 0.789; repeated measure ANOVA), which indicates that the selective deletion of Cav2.1 did not affect anxiety-like behavior.

Next, a continuous spontaneous alternation (CSA) task was conducted using a Y-maze to measure non-spatial working memory. Normal rodents tend to explore newer places compared to the previously visited locations. As shown in **Figures 2C,D**, there was no significant difference in the ratio of successful alternation between groups (control 0.68 ± 0.02%; cKO 0.68 ± 0.02%; *P* = 0.8). Moreover, control and Cav2.1 cKO mice did not differ in the total number of arm entries, which indicates that motivation to explore the environment was not significantly different between groups (control 30 ± 1.8; cKO 31.8 ± 2.8; *P* = 0.6).

Finally, mice underwent a novel object recognition task to assess non-spatial object memory (**Figures 2E,F**). Control and Cav2.1 cKO mice showed no difference in the duration of exploration of novel and familiar objects (Total duration; control 31.0 ± 5.3 s; cKO 33.4 ± 4.4 s; *P* = 0.73), suggesting no difference in motivation to explore (no significant effect of group *F*_(1)_ < 0.001, *P* = 1; significant effect of object *F*_(1)_ = 128.820, *P* < 0.001; no significant effect of group × object interaction *F*_(1)_ = 0.595, *P* = 0.447; two way ANOVA; control *P* = 0.03; cKO *P* = 0.01). Importantly, both groups preferred novel objects over familiar objects as shown in the difference index for 10 min (control 36.7 ± 6.0%; cKO 32.0 ± 5.9%; *P* = 0.58), and also in additional analyses of the first 2 and 5 min of the test phase (Supplementary Figure S2). These results suggest that both groups showed no significant difference in the ability to distinguish and remember different objects. Taken together, cKO mice did not show any indication of sensorimotor deficits in visual, vestibular and locomotion ability as well as motivation to explore as shown in the general neurological behavioral tasks.

### Cav2.1 Is Required for Accurate Spatial and Contextual Recognition

**FIGURE 3:**
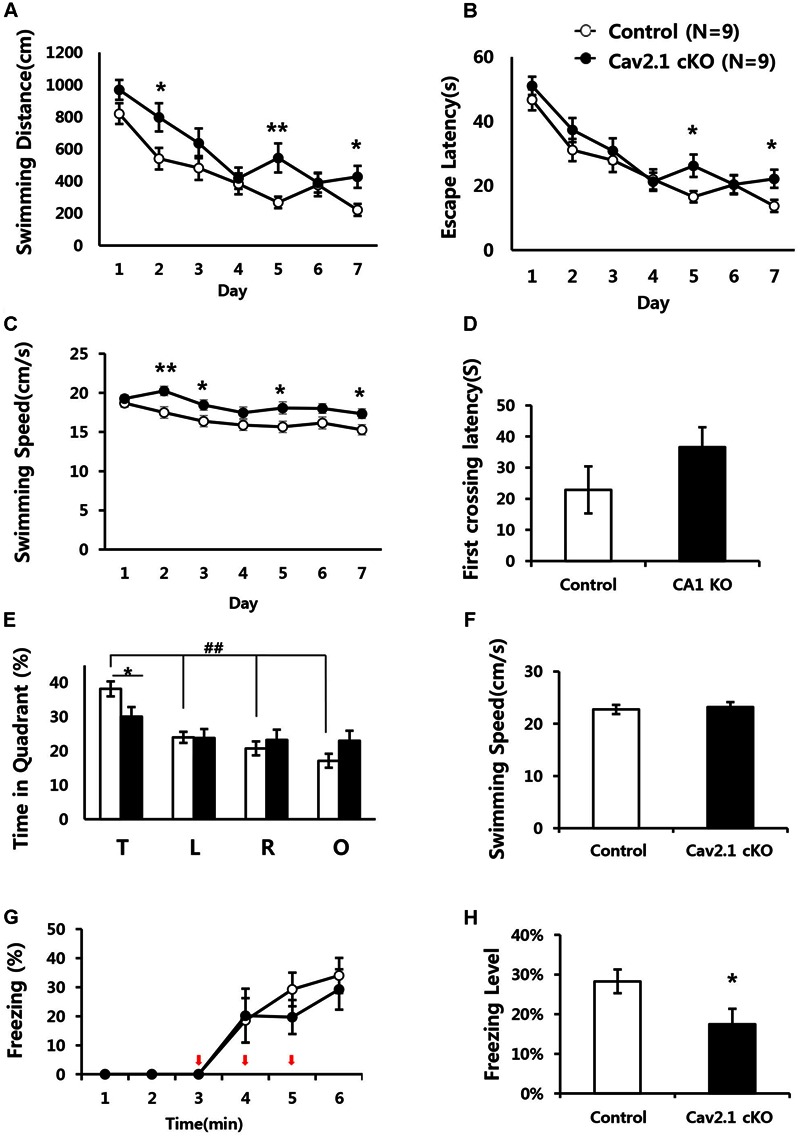
**Impaired performance in a water maze task and contextual fear conditioning task of Cav2.1 cKO mice. (A–F)** Performance in the Morris water maze task. **(A)** Total distance swimming during acquisition training day. **(B)** Latency to escape the water during acquisition training day. **(C)** Swimming speed during acquisition training day. **(D)** Latency to first crossing during probe test. **(E)** % of time spent in each quadrant swimming during probe test. **(F)** Average swimming speed during probe test. **(G–H)** Contextual fear conditioning. **(G)** % of freezing with the three foot shocks delivered during training phase. **(H)** Averaged freezing response during test phase for 10 min (an arrow indicates a foot shock, repeated measure ANOVA, Unpaired two-tailed *t*-test, ^∗^*P* < 0.05, ^∗∗^*P* < 0.01, ^##^*P* < 0.001 comparing four quadrant within groups (control, *n* = 9; cKO, *n* = 9).

Since the hippocampus plays critical roles in performing spatial navigation and contextual learning, we conducted the Morris water maze (MWM) and contextual fear conditioning tasks to determine whether Cav2.1 contributes to spatial learning and memory (**Figures 3A–F**).

In MWM, although the performance in both groups improved over the 7-day training period, Cav2.1 cKO mice showed an impairment of the performance compared to control mice as indicated by increased swimming distance (significant effect of group *F*_(1)_ = 12.196, *P* = 0.001; significant effect of day *F*_(5.443)_ = 18.658, *P* < 0.001; no significant effect of group × trial interaction effect *F*_(5.443)_ = 1.201, *P* = 0.307; repeated measure ANOVA) on several training days (2nd, 5th, 7th day, Unpaired two tailed *t*-test ^∗^*P* < 0.05, ^∗∗^*P* < 0.01). In addition, despite the increased swimming speed of cKO mice (significant effect of group *F*(1) = 25.1, *P* < 0.001; no significant effect of day ^∗^ group interaction *F*_(5.596)_ = 9.571, *P* = 0.754; repeated measure ANOVA), escape latency was significantly increased (significant effect of group *F*_(1)_ = 5.194, *P* = 0.025; significant effect of day *F*_(5.109)_ = 26.484, *P* < 0.001; no significant effect of group × trial interaction effect *F*_(5.109)_ = 0.871, *P* = 0.503; repeated measure ANOVA).

Interestingly, the impairment was more evident in the probe test conducted 24 h later with the platform removed. In **Figure 3E**, although control mice spent more time in the target quadrant relatively to the other quadrants (*F*_(3)_ = 21.60, *P* < 0.001; ANOVA with LSD *post hoc* analysis), cKO mice did not show difference in searching time across all quadrants (*F*_(3)_ = 1.38, *P* = 0.26; ANOVA with LSD *post hoc* analysis). In addition, Cav2.1 cKO mice spent less time searching in the target quadrant compared to control mice while no significant difference was observed in the other quadrants between groups (target *P* = 0.022; left *P* = 0.961; right *P* = 0.477; opposite *P* = 0.098; ANOVA with LSD *post hoc* analysis). Additional analysis of time spent in four annulus zones in quadrants showed consistent results (Supplementary Figure S3). However, the first crossing latency was not different between groups (control 22.8 ± 7.55 cKO 36.6 ± 6.35; *P* = 0.18).

Hippocampal dependent learning ability of Cav2.1 cKO was also examined through a contextual fear conditioning task (**Figures 3G,H**). To assess the ability to associate foot shocks (unconditioned stimulus) with the experimental chamber (conditioned stimulus), the mice were given three consecutive foot shocks without tones. Freezing response to the foot-shocks during the training phase was not significantly different between groups (significant effect of time *F*_(2.230)_ = 20.695, *P* < 0.001; no significant effect of group × trial interaction effect *F*_(2.230)_ = 0.452, *P* = 0.661; repeated measure ANOVA). Cav2.1 cKO mice, however, showed a significantly lower freezing response during the test phase when returned to the fear conditioning chamber 24 h after the training (control 28.3 ± 2.99%; cKO 17.5 ± 3.89; *P* = 0.046).

Taken together, these results indicate that selective deletion of Cav2.1 in the hippocampus impaired spatial and contextual learning and memory without affecting sensory, motor, emotional and motivational function, as indicated by normal swimming speed during the probe test and normal behaviors in an open field test. In addition, these results suggest that cKO mice displayed more profound problems in recognizing spatial environment than in acquiring information about the environment. This tendency is more evident in a contextual fear conditioning task than in a water maze task because a water maze task requires a greater cognitive demand.

### Cav2.1 Is Required for Accurate Recognition of CA1 Place Fields

**FIGURE 4:**
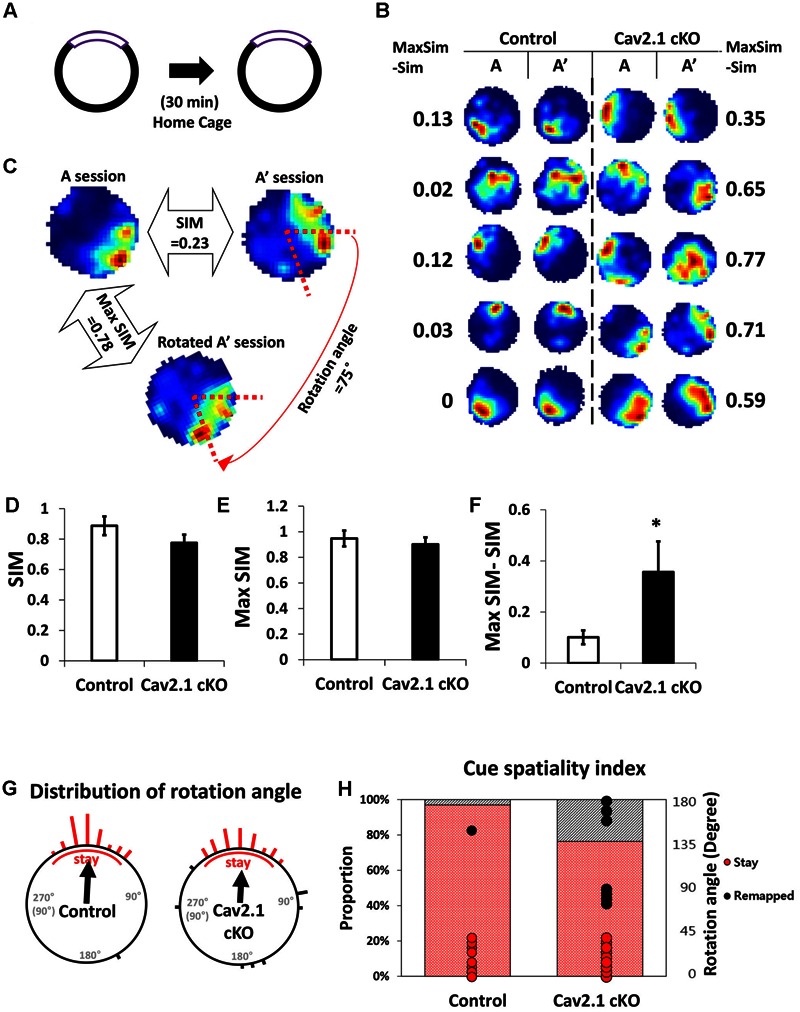
**Reproducibility of place maps between two sessions. (A)** Recording paradigm of two sessions. **(B)** Examples of place maps. **(C)** Representative examples of the similarity (SIM) values and max similarity (Max SIM) values obtained by rotating the second map. **(D)** Comparison of SIM score. **(E)** Comparison of Max SIM score. **(F)** Difference between SIM and Max SIM was increased in Cav2.1 cKO. **(G)** Distribution of rotation angles at maximum stability. Length of bars corresponds to the number of cells at each angle and an arrow indicates a mean vector. **(H)** % of ‘stay’ cells and distribution of rotation angles as shown in cue-spatiality index. Percentage of ‘stay’ cells is presented in bars and distribution of rotation angles of each cells is shown in dots (red for ‘stay,’ black for ‘remapped,’ unpaired *t*-test, Chi-square test, ^∗^*P* < 0.05).

Place cell activity was recorded to investigate the neuronal substrates underlying the observed behavioral impairments. The unit signals of CA1 pyramidal neurons from each group (33 cells from five control mice; 38 cells from five cKO mice) were consecutively recorded twice for 20 min, spaced with a 30 min break in the home cage. The recording apparatus and the orientation of the cue were maintained for both sessions in order to investigate the reproducibility of place fields when each mouse was re-exposed to the same environment after a 30 min break in the home cage (**Figures 4A,B**).

First, several properties of place rate map were compared between groups and between sessions within each group. No difference was found in intrinsic properties of place cells between groups and within sessions (Supplementary Table S1). Consistency of these properties of place cells across sessions was further confirmed by calculating the changes between sessions on individual cell basis (Supplementary Table S2). In specific, intrinsic properties of individual cells in both groups rarely changed across sessions. In particular, average place field size and coherence were similar between groups or sessions within groups. In addition, the spatial selectivity, calculated using an in-field/out-field ratio, was also similar. These results showed that place cells in both groups had similar location-dependent firing patterns with comparable firing dispersion over the recording arena, suggesting that place cells of Cav2.1 cKO mice have an intact ability to encode the exposed environment. In general, other basic characteristics of place rate map were also observed to be similar between groups (**Table 1**).

**Table 1:** General characteristics to represent space of CA1 place cells.

Property	Control	Cav2.1 cKO	*P*-value
Firing rate (Hz)	1.81 ± 0.26	1.74 ± 0.20	0.84
Mean ISI (ms)	1138 ± 184	1163 ± 193	0.92
Field size (cm^2^)	158 ± 6.39	163 ± 3.6	0.49
In field firing rate(Hz)	4.18 ± 0.57	4.02 ± 0.42	0.79
Selectivity	1.08 ± 0.05	1.05 ± 0.04	0.61
Coherence	0.97 ± 0.002	0.97 ± 0.001	0.51
Spatial information bits per s	3.15 ± 0.187	3.00 ± 0.175	0.54
Running speed(cm/s)	8.54 ± 0.14	8.18 ± 0.17	0.17

*Averaged value of place cell firing properties during two sessions. (Unpaired two-tailed *t*-test, all features was not significantly different).*

Second, a pixel-by-pixel correlation of place maps between two consecutive sessions was calculated to measure the similarity of place fields on re-visit (**Figures 4C,D**). There was a trend but no significant difference in the similarity between groups (**Figure 4D**, control 0.89 ± 0.06; cKO 0.77 ± 0.05; *P* = 0.17). In fact, the max similarity, which is the correlation score between two sessions obtained by rotating the second rate map to find the highest correlation score with that of the first, was almost identical between groups (**Figure 4E**, control 0.95 ± 0.06; cKO 0.90 ± 0.05; *P* = 0.55). In addition, the difference between max similarity and similarity scores was significantly higher in cKO mice (**Figure 4F**, control = 0.10 ± 0.03; cKO = 0.35 ± 0.12; *P* = 0.04). These results indicate that the remapping on revisit was not random in terms of location, but rather slightly mis-oriented or rotated in terms of the spatial recognition. To further investigate the pattern of difference between the similarity and max similarity of each group, analysis on distribution of rotation angle was conducted in **Figure 4G** and Supplementary Figure S4. In an analysis using circular statistics to compare the distributions, it was found that the direction of the mean vectors in both groups was similar (control = 3.8°, cKO = 3.7°; control *P* < 0.001, cKO *P* < 0.001; *v* test), meaning that the average rotation angles of both groups was homogenously distributed toward 0°. However, in mean vectors length of a scale from 0 (disperse) to 1 (focused), the control group showed 0.91 while cKO mice showed 0.69, suggesting that individual place fields of cKO mice were more dispersedly deviated compared to those of control mice. Although, the median directions of distributions in the two groups were not significantly different (*P* = 0.47; Kruskal–Wallis test), but the greater portion of place fields was observed to be rotated in Cav2.1 cKO mice. The cue spatiality index showed that a smaller portion of Cav2.1 cKO place fields stayed within 0° to ±45° on revisit [**Figure 4H**, control 32 out of 33 (97%); cKO 29 out of 38 (76%); Chi-square, *P* = 0.012] (Lee et al., 2009; Park et al., 2015). These results indicate that greater proportion of place cells of Cav2.1 cKO were unable to precisely align the place field toward the cue when re-exposed to the identical environment, i.e., precise cue spatiality. Taken all, the accuracy of spatial recognition in terms of cue-spatiality in the re-exposed environment was impaired while the general characteristics of place rate map to encode an environment were almost identical in Cav2.1 cKO mice. Overall, these results are consistent with behavioral results in that the impairments were more evident in recognition than in acquisition.

### Cav2.1 Deletion Induced Alteration in CA1 Burst Firing

**FIGURE 5:**
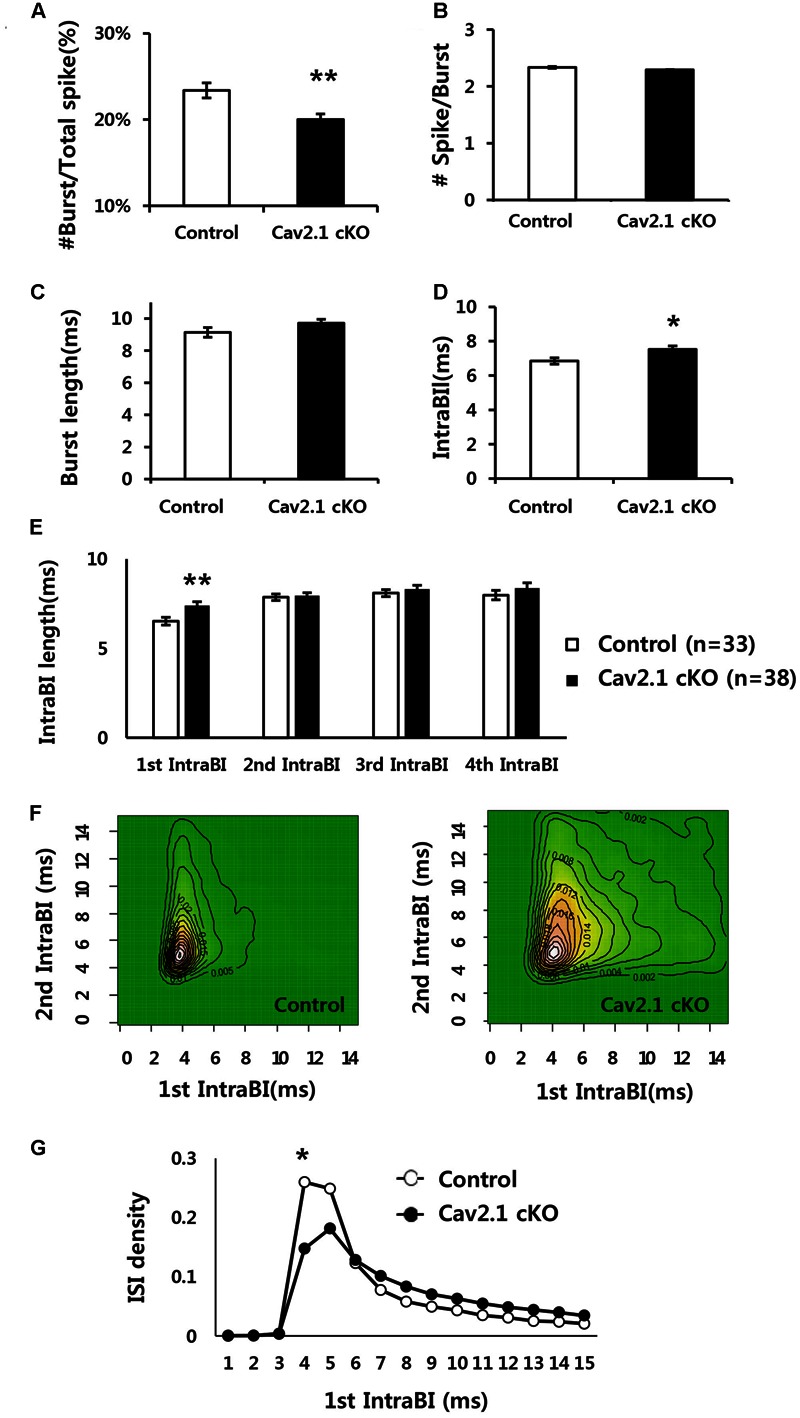
**Comparison of firing properties between control and Cav2.1 cKO groups. (A)** Ratio of burst firing to total spikes in Cav2.1 cKO was significantly decreased. **(B)** Number of spikes per burst was intact in Cav2.1 cKO. **(C)** Burst length was intact in Cav2.1 cKO. **(D)** Intra-Burst Interval (IntraBI) is significantly increased in Cav2.1 cKO. **(E)** Histogram of length of the first to fourth IntraBIs. **(F)** Joint Probability of Density (JPD) of the first and second Intra-Burst Interval (IntraBI). Control mice (left) and Cav2.1 cKO mice (right). **(G)** Distribution of inter spike interval between the first and second spike within a burst (first IntraBI) (unpaired two-tailed *t*-test, Chi-square test, ^∗^*P* < 0.05, ^∗∗^*P* < 0.01).

We also investigated the role of Cav2.1 in mediating spiking patterns and in particular, temporal components of burst firing (**Figure 5**).

First, average firing rate did not significantly differ, indicating that overall spiking in freely behaving state was not influenced by Cav2.1 (**Table 1**). However, there were significant differences in burst spiking properties. In particular, there was a significant decrease of burst ratio over total spiking in Cav2.1 cKO mice (control 23.4 ± 0.9%; cKO 20 ± 0.7%; *P* = 0.003), suggesting that Cav2.1 contributes to the generation of burst firing (**Figure 5A**). Interestingly, the temporal characteristics of spikes within a burst (**Figure 5D**) were also significantly altered as shown in prolonged spike intervals within a burst (IntraBIs) (control 6.85 ± 0.19 ms; cKO 7.53 ± 0.20 ms; *P* = 0.015). On the other hand, burst length and the number of spikes within a burst did not differ between groups (**Figures 5B,C**). The change in temporal bursting property was mainly mediated by the prolonged interval between the first spike and the second spike within a burst, i.e., the first IntraBI (first IntraBI *P* = 0.007; second IntraBI *P* = 0.796; third IntraBI *P* = 0.487; fourth IntraBI *P* = 0.244; ANOVA with LSD *post hoc* analysis), as shown in **Figure 5E** as well as in the joint probability density (JPD) analysis (see Materials and Methods, **Figure 5F**). The distribution of the first IntraBI was more dispersed while the second IntraBI remained intact in Cav2.1 cKO (**Figure 5F**). This result was also confirmed by the comparison of the density distribution of the first IntraBI (**Figure 5G**), which was more dispersed temporally in Cav2.1 cKO. In addition, the power spectral density of theta frequency (6–10 Hz) using spike train data (Kim et al., 2015) revealed there was no remarkable difference between groups (Supplementary Figure S5). These results suggest that the ablation of Cav2.1 of the hippocampal regions interferes with the first interval only, but not other intervals, resulting in intact theta oscillation, altered properties of the burst generation and the temporal integration.

Overall, this study aimed to investigate the role of pyramidal Cav2.1 (P/Q type calcium channel) on hippocampal-dependent spatial memory from neuronal to behavioral levels using behaving mice lacking Cav2.1 in the hippocampus. Results consistently demonstrated that conditional ablation of Cav2.1 induced alterations in burst spiking pattern, recognition of place fields and hippocampal-dependent learning and memory.

## Discussion

### Declining Behavioral Performance in Spatial Recognition

The performance of cKO mice was significantly impaired in hippocampal-dependent behavioral tasks but not in general tasks. Behavioral impairments might have originated from moderate Cav2.1 deletions of other forebrain regions beside the hippocampus. However, all behavioral impairments of cKO were rather shown in hippocampal dependent tasks such as water maze and contextual fear conditioning tasks, but not in other tasks including Y-maze, open field and object recognition tasks, suggesting that behavioral roles of Cav2.1 in other forebrain regions, excluding the hippocampus, were considerably limited. Despite the high density of Cav2.1 in hippocampal regions, the deletion of Cav2.1 caused rather mild impairments during the acquisition of the water maze task but caused profound deficits during the probe test. While moderate impairment was shown during the acquisition of a water maze task, the performance in a contextual fear conditioning task was intact during the acquisition. In addition, deletion of Cav2.1 in the entorhinal cortex, which is a major afferent to the hippocampus, did not directly influence the firing activity of CA1 neurons unlike in other studies reporting that malfunction of the entorhinal cortex caused reductions in either firing rate or spatial information score of CA1 place cells (Brun et al., 2008; Van Cauter et al., 2008; Zhao et al., 2016). This suggests that the effect of moderate deletion of Cav2.1 in the entorhinal cortex was minimal on the place cell activity. Nonetheless, our results suggest the critical involvement of Cav2.1 in processing spatial recognition related to hippocampal-dependent behavioral performance, which is comparable with the observed changes in CA1 place cell.

### Impaired Accuracy of Spatial Recognition of CA1 Place Fields in Cav2.1 cKO

Ablation of Cav2.1 in CA1 pyramidal cells did not induce any significant changes in intrinsic properties of spatial representation in terms of CA1 place cell activity, indicating that the ability of cKO place cells in learning an environment was intact. In fact, the malfunction of head-direction system and ideothetic cue processing may interfere place field stability (Gothard et al., 1996; Calton et al., 2003). However, size and coherence of place cells remained intact within one session (implying intact stability and no shifting) in cKO, while rotational remapping occurred only between two sessions. Besides, other brain regions related to those system (i.e., thalamus), other than hippocampus, were not expected to express Cre recombinase to have intact Cav2.1 expression.

In details, greater portion of place cells in Cav2.1 cKO showed mis-alignment between two place fields toward the cue when mice were re-exposed to the same environment. Although, the effect of selective deletion of Cav2.1 is rather partial, more specific ablation on the whole hippocampus could have revealed more evident role of Cav2.1 in place cell activity in relation with spatial/contextual behaviors. Indeed, place fields from two consecutive sessions from Cav2.1 cKO became similar to the level of control place fields when one of the place fields was rotated, as shown in **Figure 4F**. In other words, Cav2.1 deletion prevented place cells from precisely recalling the topological spatial representation based on the cue. These results suggest that Cav2.1 in CA1 plays an important role in precisely recognizing spatial relations in the same environment at a neuronal level, which is comparable with the role of other cellular molecules in CA1 in terms of spatial reproducibility (Cacucci et al., 2007).

### Effects in Burst Firing Pattern

Our results demonstrate that Cav2.1 take part in the generation of burst firing in CA1 as implicated by decreased proportion of burst in behaving mice conditionally lacking Cav2.1. It is possible that the prolongation of the first IntraBI might increase probability of failing burst generation to some extent. Furthermore, abnormal IntraBI induced by Cav2.1 deletion also provides substantial evidence that Cav2.1 is involved in modulating the temporal component of burst firing.

Although, the mechanisms through which Cav2.1 may modulate burst firing in the hippocampus are not well-established, it might contribute to shape intrinsic firing properties such as afterhyperpolarization (AHP) to modulate firing rate of burst spikes by activating various channels such as Iberiotoxin-sensitive large conductance Ca^2+^-dependent K^+^ (BK) and Small-conductance calcium-activated potassium channels (SK2) (Magee and Carruth, 1999). Another possibility is that it can shape afterdepolarization (ADP), which is also known to be generated by R-type calcium channel (a subtype of the Cav2 family) in the hippocampus, to increase the probability of consecutive spike generation (Magee and Carruth, 1999; Metz et al., 2005). In addition, Cav2.1 is also thought to play a role in modulating burst firing through dendritic Ca^2+^ spike as shown in slice experiments using Cav2.1 transgenic mice (Magee and Carruth, 1999; Ovsepian and Friel, 2008). In fact, many studies have shown that dendritic spikes require NMDA channels and VGCCs that are involved in synaptic plasticity and long term potentiation, and that combined activation of NMDA channels and VGCCs are required for burst firing *in vivo* (Grienberger et al., 2014). Considering the previous studies, it is possible for dendritic Ca^2+^ influx via Cav2.1 to play a role in learning and memory in collaboration with NMDA channels by modulating bursting in that NMDA have been known to be involved in learning an memory both *in vitro* and *in vivo* studies (Cui et al., 2004; Moosmang et al., 2005; Place et al., 2012).

### Altered Burst Properties and Spatial Learning and Memory

Our study showed that hippocampal deletion of Cav2.1 altered both burst properties and cue spatiality of CA1 place fields, but the direct modulatory mechanism between burst firing and spatial learning is unclear. Several studies have attempted to reveal properties of burst firing in the hippocampus and its distinctive role at the behavioral level. For example, the synaptotagmin-1 knock-down mice, which abolishes neurotransmitter release evoked by a selective firing code, was capable of learning in a contextual learning task but failed to accurately recall such memory (Xu et al., 2012). Their studies are relevant with our results in that Cav2.1 ablation induced alteration in burst firing as well as inaccuracy in spatial recollection without affecting global encoding ability. In addition, other studies have suggested the functional correlation between burst firing property and learning ability using knockout mice models. For examples, HCN1 (hyperpolarization-activated cation channel) knockout mice displaying improvement in learning and stable spatial representation of place cells also showed higher proportion of burst firing (Hussaini et al., 2011). In contrast, α-CamK2 knockout mice with severe impairments in both behavior and spatial representation ability of place cells showed substantial reduction of CA1 burst firing (Cho et al., 2012). Furthermore, the present study provides potential evidence of more specific correlation between CA1 pyramidal bursting and spatial behavior in that hippocampal deletion of Cav2.1 caused the partial disruption in both bursting generation and recollection of place fields along with comparable deficits shown in recognition of hippocampal dependent behavioral tasks.

Taken together, we believe that these results present substantially useful evidence that can provide a potential link between hippocampal dependent spatial/contextual behaviors, place cell activity and Cav2.1 channel in general, which would facilitate the further studies on interactive mechanisms underlying electrophysiological and behavioral effects of Cav2.1.

## Author Contributions

DJ and JC designed the experiments. JC supervised the project. DJ, YH, HR performed experiments. MK and KS provided Cav2.1 floxed mice line. DJ and JC analyzed the data. DJ and JC wrote the manuscript.

## Conflict of Interest Statement

The authors declare that the research was conducted in the absence of any commercial or financial relationships that could be construed as a potential conflict of interest. The reviewer ES and handling Editor declared their shared affiliation, and the handling Editor states that the process nevertheless met the standards of a fair and objective review.

## References

[B1] Barbarese E. Ifrim M. F. Hsieh L. Guo C. Tatavarty V. Maggipinto M. J. (2013). Conditional knockout of tumor overexpressed gene in mouse neurons affects RNA granule assembly, granule translation, LTP and short term habituation. *PLoS ONE* 8:e69989 10.1371/journal.pone.0069989 PMC3735573 23936366

[B2] Brigman J. L. Daut R. A. Wright T. Gunduz-Cinar O. Graybeal C. Davis M. I. (2013). GluN2B in corticostriatal circuits governs choice learning and choice shifting. *Nat. Neurosci.* 16 1101–1110. 10.1038/nn.3457 23831965 PMC3725191

[B3] Broadbent N. J. Gaskin S. Squire L. R. Clark R. E. (2010). Object recognition memory and the rodent hippocampus. *Learn. Mem.* 17 5–11. 10.1101/lm.1650110 20028732 PMC2807177

[B4] Brun V. H. Leutgeb S. Wu H.-Q. Schwarcz R. Witter M. P. Moser E. I. (2008). Impaired spatial representation in CA1 after lesion of direct input from entorhinal cortex. *Neuron* 57 290–302. 10.1016/j.neuron.2007.11.034 18215625

[B5] Cacucci F. Wills T. J. Lever C. Giese K. P. O’Keefe J. (2007). Experience-dependent increase in CA1 place cell spatial information, but not spatial reproducibility, is dependent on the autophosphorylation of the alpha-isoform of the calcium/calmodulin-dependent protein kinase II. *J. Neurosci.* 27 7854–7859. 10.1523/JNEUROSCI.1704-07.2007 17634379 PMC2680063

[B6] Calton J. L. Stackman R. W. Goodridge J. P. Archey W. B. Dudchenko P. A. Taube J. S. (2003). Hippocampal place cell instability after lesions of the head direction cell network. *J. Neurosci.* 23 9719–9731. 14585999 10.1523/JNEUROSCI.23-30-09719.2003 PMC6740880

[B7] Catterall W. A. Few A. P. (2008). Calcium channel regulation and presynaptic plasticity. *Neuron* 59 882–901. 10.1016/j.neuron.2008.09.005 18817729

[B8] Catterall W. A. Leal K. Nanou E. (2013). Calcium channels and short-term synaptic plasticity. *J. Biol. Chem.* 288 10742–10749. 10.1074/jbc.R112.411645 23400776 PMC3624454

[B9] Cho J. Bhatt R. Elgersma Y. Silva A. J. (2012). alpha-Calcium calmodulin kinase II modulates the temporal structure of hippocampal bursting patterns. *PLoS ONE* 7:e31649 10.1371/journal.pone.0031649 PMC3282754 22363696

[B10] Cui Z. Wang H. Tan Y. Zaia K. A. Zhang S. Tsien J. Z. (2004). Inducible and reversible NR1 knockout reveals crucial role of the NMDA receptor in preserving remote memories in the brain. *Neuron* 41 781–793. 10.1016/S0896-6273(04)00072-8 15003177

[B11] D’Hooge R. De Deyn P. P. (2001). Applications of the Morris water maze in the study of learning and memory. *Brain Res. Rev.* 36 60–90. 10.1016/S0165-0173(01)00067-4 11516773

[B12] Dix S. L. Aggleton J. P. (1999). Extending the spontaneous preference test of recognition: evidence of object-location and object-context recognition. *Behav. Brain Res.* 99 191–200. 10.1016/S0166-4328(98)00079-5 10512585

[B13] Gamelli A. E. McKinney B. C. White J. A. Murphy G. G. (2011). Deletion of the L-type calcium channel Ca(V) 1.3 but not Ca(V) 1.2 results in a diminished sAHP in mouse CA1 pyramidal neurons. *Hippocampus* 21 133–141. 10.1002/hipo.20728 20014384 PMC2891900

[B14] Gothard K. M. Skaggs W. E. McNaughton B. L. (1996). Dynamics of mismatch correction in the hippocampal ensemble code for space: interaction between path integration and environmental cues. *J. Neurosci.* 16 8027–8040. 8987829 10.1523/JNEUROSCI.16-24-08027.1996 PMC6579211

[B15] Grienberger C. Chen X. Konnerth A. (2014). NMDA receptor-dependent multidendrite Ca(2+) spikes required for hippocampal burst firing in vivo. *Neuron* 81 1274–1281. 10.1016/j.neuron.2014.01.014 24560703

[B16] Harris K. D. Hirase H. Leinekugel X. Henze D. A. Buzsaki G. (2001). Temporal interaction between single spikes and complex spike bursts in hippocampal pyramidal cells. *Neuron* 32 141–149. 10.1016/S0896-6273(01)00447-0 11604145

[B17] Hashimoto K. Tsujita M. Miyazaki T. Kitamura K. Yamazaki M. Shin H. S. (2011). Postsynaptic P/Q-type Ca2+ channel in Purkinje cell mediates synaptic competition and elimination in developing cerebellum. *Proc. Natl. Acad. Sci. U.S.A.* 108 9987–9992. 10.1073/pnas.1101488108 21628556 PMC3116426

[B18] Hillman D. Chen S. Aung T. T. Cherksey B. Sugimori M. Llinas R. R. (1991). Localization of P-type calcium channels in the central-nervous-system. *Proc. Natl. Acad. Sci. U.S.A.* 88 7076–7080. 10.1073/pnas.88.16.7076 1651493 PMC52236

[B19] Hussaini S. A. Kempadoo K. A. Thuault S. J. Siegelbaum S. A. Kandel E. R. (2011). Increased size and stability of CA1 and CA3 place fields in HCN1 knockout mice. *Neuron* 72 643–653. 10.1016/j.neuron.2011.09.007 22099465 PMC4435580

[B20] Huxter J. Burgess N. O’Keefe J. (2003). Independent rate and temporal coding in hippocampal pyramidal cells. *Nature* 425 828–832. 10.1038/nature02058 14574410 PMC2677642

[B21] Izhikevich E. M. Desai N. S. Walcott E. C. Hoppensteadt F. C. (2003). Bursts as a unit of neural information: selective communication via resonance. *Trends Neurosci.* 26 161–167. 10.1016/S0166-2236(03)00034-1 12591219

[B22] Jun K. Piedras-Renteria E. S. Smith S. M. Wheeler D. B. Lee S. B. Lee T. G. (1999). Ablation of P/Q-type Ca(2+) channel currents, altered synaptic transmission, and progressive ataxia in mice lacking the alpha(1A)-subunit. *Proc. Natl. Acad. Sci. U.S.A.* 96 15245–15250. 10.1073/pnas.96.26.15245 10611370 PMC24805

[B23] Kim E. J. Park M. Kong M.-S. Park S. G. Cho J. Kim J. J. (2015). Alterations of hippocampal place cells in foraging rats facing a “predatory”. *Threat. Curr. Biol.* 25 1362–1367. 10.1016/j.cub.2015.03.048 25891402 PMC4439350

[B24] Kim H. V. Kim H. Y. Ehrlich H. Y. Choi S. Y. Kim D. J. Kim Y. (2013). Amelioration of Alzheimer’s disease by neuroprotective effect of sulforaphane in animal model. *Amyloid* 20 7–12. 10.3109/13506129.2012.751367 23253046

[B25] Kim J. J. Lee H. J. Welday A. C. Song E. Cho J. Sharp P. E. (2007). Stress-induced alterations in hippocampal plasticity, place cells, and spatial memory. *Proc. Natl. Acad. Sci. U.S.A.* 104 18297–18302. 10.1073/pnas.0708644104 17984057 PMC2084337

[B26] Koch H. Zanella S. Elsen G. E. Smith L. Doi A. Garcia A. J. (2013). Stable respiratory activity requires both P/Q-type and N-type voltage-gated calcium channels. *J. Neurosci.* 33 3633–3645. 10.1523/JNEUROSCI.6390-11.2013 23426690 PMC3652398

[B27] Koh H. Y. Kim D. Lee J. Lee S. Shin H. S. (2008). Deficits in social behavior and sensorimotor gating in mice lacking phospholipase Cbeta1. *Genes Brain Behav.* 7 120–128. 10.1111/j.1601-183X.2007.00351.x 17696993

[B28] Lee J. W. Kim W. R. Sun W. Jung M. W. (2009). Role of dentate gyrus in aligning internal spatial map to external landmark. *Learn. Mem.* 16 530–536. 10.1101/lm.1483709 19706836

[B29] Lisman J. E. (1997). Bursts as a unit of neural information: making unreliable synapses reliable. *Trends Neurosci.* 20 38–43. 10.1016/S0166-2236(96)10070-9 9004418

[B30] Liu S. Friel D. D. (2008). Impact of the leaner P/Q-type Ca2+ channel mutation on excitatory synaptic transmission in cerebellar Purkinje cells. *J. Physiol.* 586 4501–4515. 10.1113/jphysiol.2008.156232 18669535 PMC2614031

[B31] Llinas R. R. Choi S. Urbano F. J. Shin H. S. (2007). Gamma-band deficiency and abnormal thalamocortical activity in P/Q-type channel mutant mice. *Proc. Natl. Acad. Sci. U.S.A.* 104 17819–17824. 10.1073/pnas.0707945104 17968008 PMC2077027

[B32] Magee J. C. Carruth M. (1999). Dendritic voltage-gated ion channels regulate the action potential firing mode of hippocampal CA1 pyramidal neurons. *J. Neurophysiol.* 82 1895–1901. 10515978 10.1152/jn.1999.82.4.1895

[B33] Mallmann R. T. Elgueta C. Sleman F. Castonguay J. Wilmes T. van den Maagdenberg A. (2013). Ablation of Ca(V)2.1 voltage-gated Ca(2)(+) channels in mouse forebrain generates multiple cognitive impairments. *PLoS One* 8:e78598 10.1371/journal.pone.0078598 PMC3814415 24205277

[B34] Metz A. E. Jarsky T. Martina M. Spruston N. (2005). R-type calcium channels contribute to afterdepolarization and bursting in hippocampal CA1 pyramidal neurons. *J. Neurosci.* 25 5763–5773. 10.1523/JNEUROSCI.0624-05.2005 15958743 PMC6724888

[B35] Moosmang S. Haider N. Klugbauer N. Adelsberger H. Langwieser N. Muller J. (2005). Role of hippocampal Cav1.2 *Ca*2+ channels in NMDA receptor-independent synaptic plasticity and spatial memory. *J Neurosci.* 25 9883–9892. 10.1523/JNEUROSCI.1531-05.2005 16251435 PMC6725564

[B36] Muller R. U. Kubie J. L. (1987). The effects of changes in the environment on the spatial firing of hippocampal complex-spike cells. *J. Neurosci.* 7 1951–1968. 3612226 10.1523/JNEUROSCI.07-07-01951.1987 PMC6568940

[B37] Nanou E. Sullivan J. M. Scheuer T. Catterall W. A. (2016). Calcium sensor regulation of the CaV2. 1 Ca2+ channel contributes to short-term synaptic plasticity in hippocampal neurons. *Proc. Natl. Acad. Sci.* 113 1062–1067. 10.1073/pnas.1524636113 26755594 PMC4743814

[B38] O’Keefe J. (1979). A review of the hippocampal place cells. *Prog. Neurobiol.* 13 419–439. 10.1016/0301-0082(79)90005-4 396576

[B39] O’Keefe J. Dostrovsky J. (1971). The hippocampus as a spatial map. Preliminary evidence from unit activity in the freely-moving rat. *Brain Res.* 34 171–175. 10.1016/0006-8993(71)90358-1 5124915

[B40] Ovsepian S. V. Friel D. D. (2008). The leaner P/Q-type calcium channel mutation renders cerebellar Purkinje neurons hyper-excitable and eliminates Ca2+-Na+ spike bursts. *Eur. J. Neurosci.* 27 93–103. 10.1111/j.1460-9568.2007.05998.x 18093175

[B41] Park M. Kim C. H. Jo S. Kim E. J. Rhim H. Lee C. J. (2015). Chronic stress alters spatial representation and bursting patterns of place cells in behaving mice. *Sci. Rep.* 5 16235 10.1038/srep16235 PMC4637823 26548337

[B42] Place R. Lykken C. Beer Z. Suh J. McHugh T. J. Tonegawa S. (2012). NMDA signaling in CA1 mediates selectively the spatial component of episodic memory. *Learn. Mem.* 19 164–169. 10.1101/lm.025254.111 22419815 PMC3312619

[B43] Rudy J. W. Huff N. C. Matus-Amat P. (2004). Understanding contextual fear conditioning: insights from a two-process model. *Neurosci. Biobehav. Rev.* 28 675–685. 10.1016/j.neubiorev.2004.09.004 15555677

[B44] Ryu H. Lee J. Hagerty S. W. Soh B. Y. McAlpin S. E. Cormier K. A. (2006). ESET/SETDB1 gene expression and histone H3 (K9) trimethylation in Huntington’s disease. *Proc. Natl. Acad. Sci. U.S.A.* 103 19176–19181. 10.1073/pnas.0606373103 17142323 PMC1748195

[B45] Skaggs W. E. McNaughton B. L. Gothard K. M. Markus E. J. (1993). “An information-theoretic approach to deciphering the hippocampal code,” in *Advances in Neural Information Processing 5* eds Hanson S. J. Cowan J. D. Giles C. L. (San Mateo, CA: Morgan Kaufmann Pub).

[B46] Tsien J. Z. Chen D. F. Gerber D. Tom C. Mercer E. H. Anderson D. J. (1996). Subregion- and cell type-restricted gene knockout in mouse brain. *Cell* 87 1317–1326. 10.1016/S0092-8674(00)81826-7 8980237

[B47] Van Cauter T. Poucet B. Save E. (2008). Unstable CA1 place cell representation in rats with entorhinal cortex lesions. *Eur. J. Neurosci.* 27 1933–1946. 10.1111/j.1460-9568.2008.06158.x 18412614

[B48] White J. A. McKinney B. C. John M. C. Powers P. A. Kamp T. J. Murphy G. G. (2008). Conditional forebrain deletion of the L-type calcium channel Ca V 1.2 disrupts remote spatial memories in mice. *Learn. Mem.* 15 1–5. 10.1101/lm.773208 18174367

[B49] Xu W. Morishita W. Buckmaster P. S. Pang Z. P. Malenka R. C. Sudhof T. C. (2012). Distinct neuronal coding schemes in memory revealed by selective erasure of fast synchronous synaptic transmission. *Neuron* 73 990–1001. 10.1016/j.neuron.2011.12.036 22405208 PMC3319466

[B50] Zhao R. Grunke S. D. Keralapurath M. M. Yetman M. J. Lam A. Lee T.-C. (2016). Impaired recall of positional memory following chemogenetic disruption of place field stability. *Cell Rep.* 16 793–804. 10.1016/j.celrep.2016.06.032 27373150 PMC4956499

