# Corrigendum: Conditional Knockout of Cav2.1 Disrupts the Accuracy of Spatial Recognition of CA1 Place Cells and Spatial/Contextual Recognition Behavior

**DOI:** 10.3389/fnbeh.2017.00242

**Published:** 2017-12-08

**Authors:** Dahee Jung, Yu J. Hwang, Hoon Ryu, Masanobu Kano, Kenji Sakimura, Jeiwon Cho

**Affiliations:** ^1^Center for Neuroscience, Korea Institute of Science and Technology, Seoul, South Korea; ^2^Neuroscience Program, Korea University of Science and Technology, Daejeon, South Korea; ^3^Centre for Neuromedicine, Brain Science Institute, Korea Institute of Science and Technology, Seoul, South Korea; ^4^VA Boston Healthcare System, Department of Neurology and Boston University Alzheimer's Disease Centre, Boston University School of Medicine, Boston, MA, United States; ^5^Department of Neurophysiology, Graduate School of Medicine, University of Tokyo, Tokyo, Japan; ^6^Department of Cellular Neurobiology, Brain Research Institute, Niigata University, Niigata, Japan

**Keywords:** P/Q type calcium channels, burst, hippocampus, place cell, learning and memory

In the original article, there were errors on citation. The statement on the mouse line from Mallmann's paper (Mallmann et al., 2013) was found to be irrelevant to our work and conclusion; thus the statement “Although a recent study circumvented the lethal phenotype by using the Cre-loxP system under the control of the NEX promoter to delete Cav2.1 in the neocortex, the mice still displayed substantial emotional impairments including anxiety and seizure. These affective changes observed in this transgenic mice line may have interfered with their performance in learning and memory tasks, therefore, results could be inconclusive in its attempt to identify the role of Cav2.1 in spatial learning and memory and hippocampal place cell activity” in the introduction section should be eliminated from this article.

In addition, the citation in the discussion section was misplaced at the end of statement to mislead information on specific subtype of calcium channels as following “Considering the previous studies, it is possible for dendritic Ca^2+^ influx via Cav2.1 to play a role in learning and memory in collaboration with NMDA channels by modulating bursting in that NMDA have been known to be involved in learning and memory both in vitro and in vivo studies (Cui et al., 2004; Moosmang et al., 2005; Place et al., 2012).” Therefore, the citation should be replaced to the appropriate place “Considering the previous studies (Cui et al., 2004; Moosmang et al., 2005; Place et al., 2012).”

The authors apologize for this error and state that this does not change the scientific conclusions of the article in any way.

The original article has been updated.

## Conflict of interest statement

The authors declare that the research was conducted in the absence of any commercial or financial relationships that could be construed as a potential conflict of interest.

## References

[B1] Cui Z. Wang H. Tan Y. Zaia K. A. Zhang S. Tsien J. Z. (2004). Inducible and reversible NR1 knockout reveals crucial role of the NMDA receptor in preserving remote memories in the brain. Neuron 41, 781–793. 10.1016/S0896-6273(04)00072-8 15003177

[B2] Mallmann R. T. Elgueta C. Sleman F. Castonguay J. Wilmes T. van den Maagdenberg A. (2013). Ablation of Ca_V_2.1 voltage-gated Ca^2+^ channels in mouse forebrain generates multiple cognitive impairments. PLoS One 8:e78598 10.1371/journal.pone.0078598 24205277 PMC3814415

[B3] Moosmang S. Haider N. Klugbauer N. Adelsberger H. Langwieser N. Muller J.. (2005). Role of hippocampal Ca_v_1.2 Ca^2+^ channels in NMDA receptor-independent synaptic plasticity and spatial memory. J Neurosci. 25, 9883–9892. 10.1523/JNEUROSCI.1531-05.2005 16251435 PMC6725564

[B4] Place R. Lykken C. Beer Z. Suh J. McHugh T. J. Tonegawa S.. (2012). NMDA signaling in CA1 mediates selectively the spatial component of episodic memory. Learn. Mem. 19, 164–169. 10.1101/lm.025254.111 22419815 PMC3312619

