# Supplementary material for: Conditional Knockout of Cav2.1 Disrupts the Accuracy of Spatial Recognition of CA1 Place Cells and Spatial/Contextual Recognition Behavior
Source: Front Behav Neurosci. 2016 Nov 3;10:214. doi: 10.3389/fnbeh.2016.00214 (PMC5093114; doi:10.3389/fnbeh.2016.00214)
Supplement: Supplementary file 1 [file Data_Sheet_1.pdf]

Supplementary Material

Conditional knockout of Cav2.1 disrupts the accuracy of spatial recognition of CA1 place cells and spatial/contextual recognition behavior.

Dahee Jung[1,4], Yu Jin Hwang[2], Hoon Ryu[2,3,], Masanobu Kano[5], Kenji Sakimura[6], Jeiwon Cho[1,4*]

[1] Center for Neuroscience, Korea Institute of Science and Technology, 5 Hwarang-ro 14-gil, Seongbuk-gu, Seoul 136-791, Korea

[2] Centre for Neuromedicine, Brain Science Institute, Korea Institute of Science and Technology, Seoul, 136-791, South Korea

[3]VA Boston Healthcare System, Department of Neurology and Boston University Alzheimer's Disease Centre, Boston University School of Medicine, Boston, MA 02118, USA

[4] Neuroscience Program, Korea University of Science & Technology, 217 Gajeong-ro, Daejeon 34113, Korea

[5] Department of Neurophysiology, Graduate School of Medicine, University of Tokyo, Tokyo 113-0033, Japan

[6] Department of Cellular Neurobiology, Brain Research Institute. Niigata University, Niigata 951-8585, Japan

*** Correspondence:**
Jeiwon Cho, Center for Neuroscience, Korea Institute of Science and Technology, 5 Hwarang-ro 14-gil, Seongbuk-gu, Seoul 136-791, Korea

Phone: 82-2-958-6955

Email: jeiwon@kist.re.kr

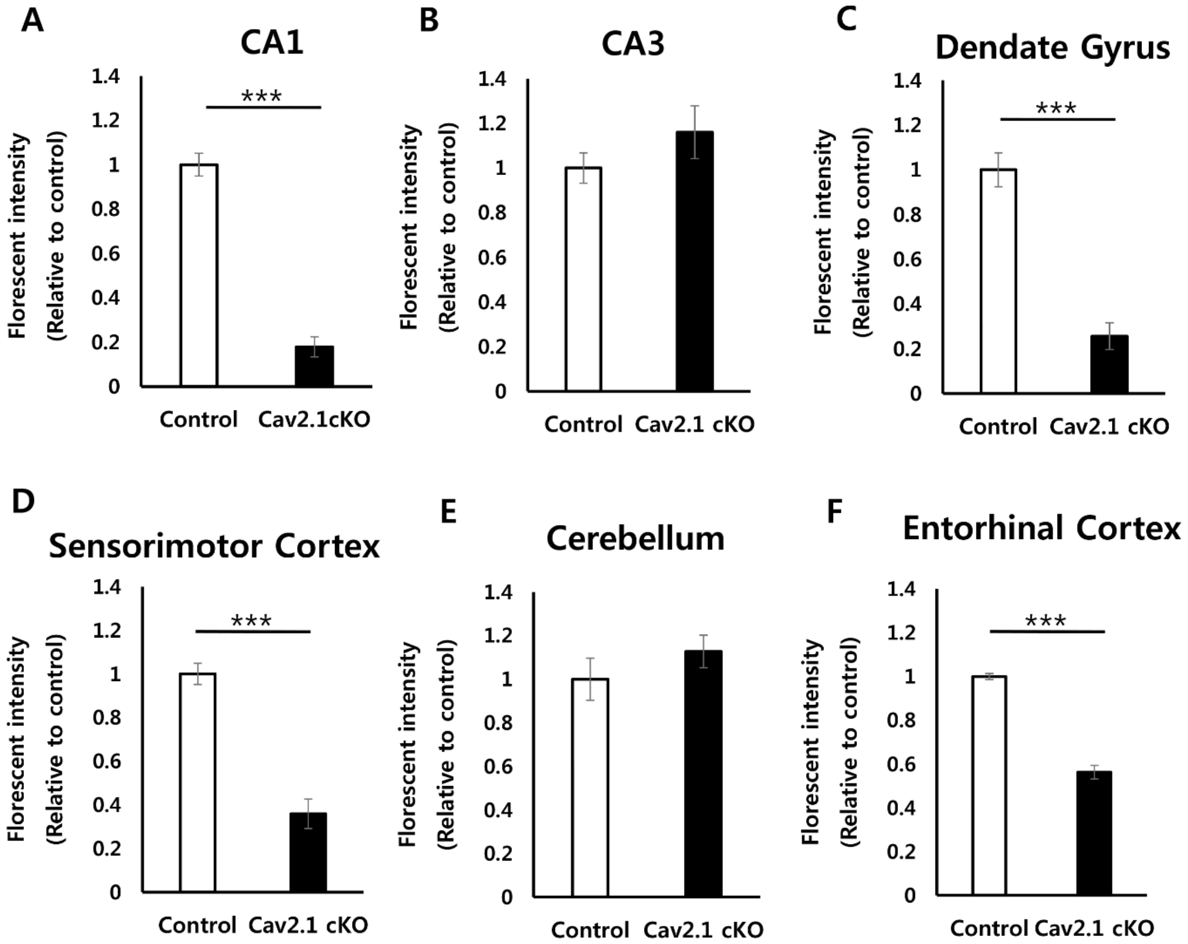

Supplementary Figure S1. Quantification of Cav2.1 expression by comparing the mean fluorescent intensity. (A) CA1 (Control=1±0.051; cKO=0.17+0.045; P<0.001) (B) CA3 (Control=1±0.067; cKO=1.15+0.118; P=0.25) (C) Dendate gyrus (Control=1±0.076; cKO=0.25+0.059; P<0.001) (D) Sensorimotor cortex (Control=1±0.048; cKO=0.36+0.067; P<0.001) (E) Cerebellum (Control=1±0.096; cKO=1.12+0.074; P=0.29) (F) Entorhinal cortex (Control=1±0.013; cKO=0.56±0.31; P<0.001) ***P<0.001

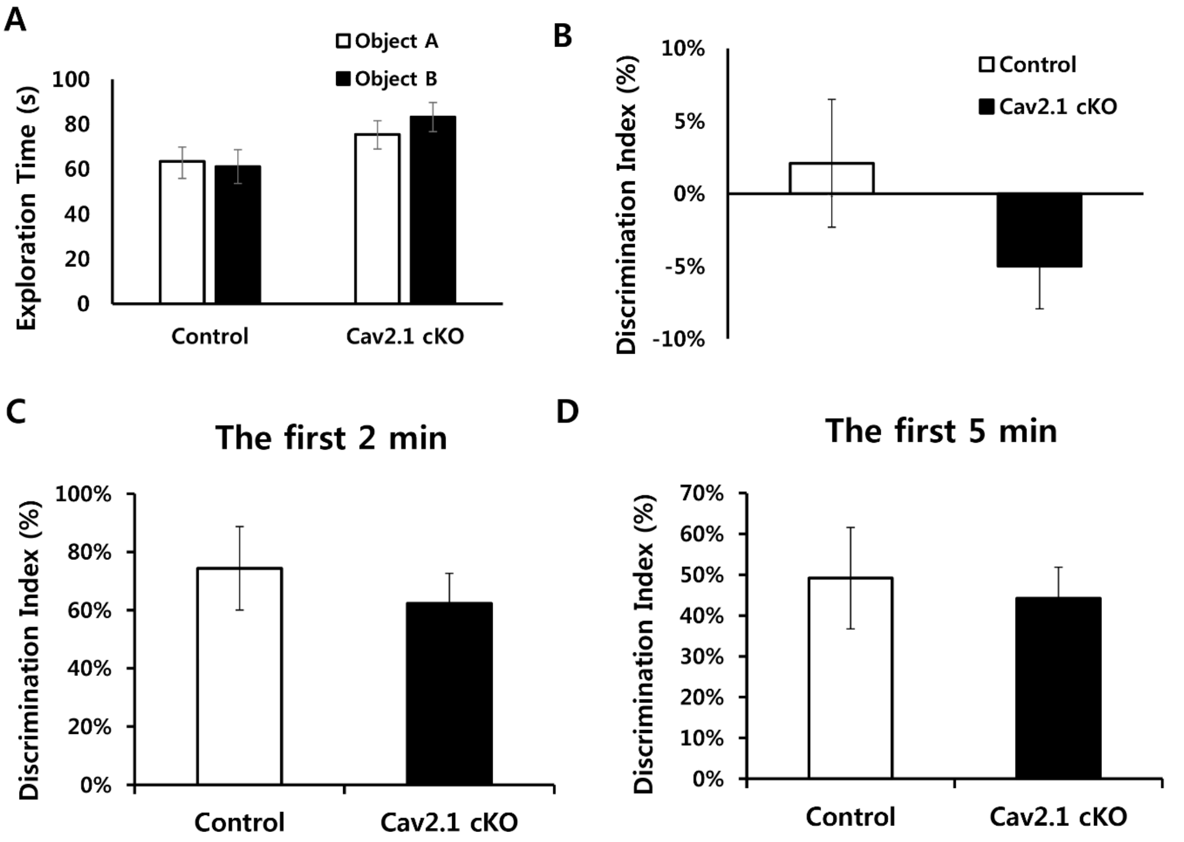

Supplementary Figure S2. Novel object recognition task. (A,B) During training phase. (A) Both groups did not show any preference over the two objects (Control P=0.83; cKO P=0.39). (B) Discrimination indices were similar in both groups (Control 0.02±0.044 ; cKO -0.05±0.030; P=0.21). (C,D) During task phase. (C) Discrimination index during the first 2 min (Control 0.74±0.143 ; cKO 0.62±0.102; P=0.51). (D) Discrimination index during the first 5 min (Control 0.49±0.124 ; cKO 0.44±0.076; P=0.74).

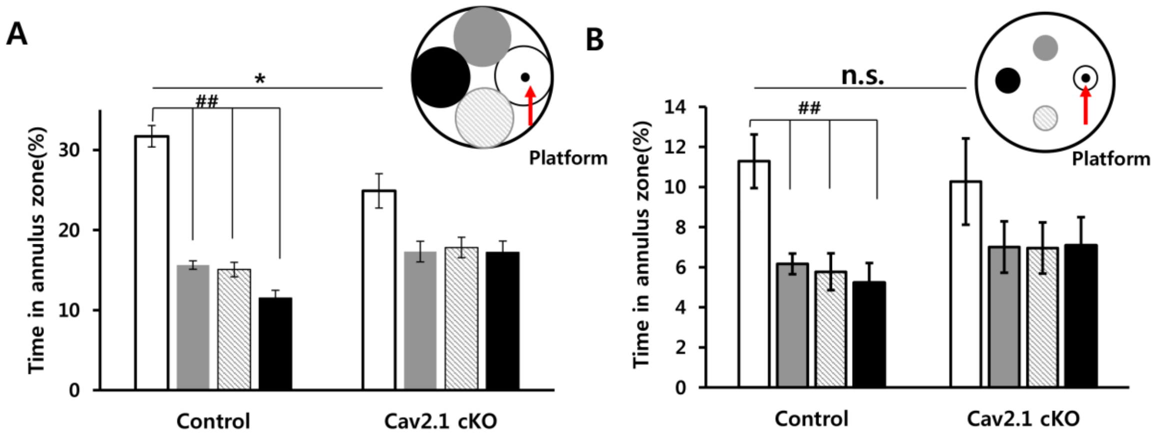

Supplementary Figure S3. Swimming time (%) in annulus zone during the probe test of a water maze task. (A)Time spent in annulus zone (quadrant size) during probe test. In control group, time in target annulus was significantly different from others ($F_{(3)}$=8.15 P<0.001; ANOVA with LSD post hoc analysis), while cKO group show similar extent of spending time in all zone ($F_{(3)}$=1.07 P=0.085; ANOVA with LST post hoc analysis). (B)Time spent in annulus zone (3x platform size) during probe test. In control group, time in target annulus was significantly different from others ($F_{(3)}$=8.15 P<0.001; ANOVA with LSD post hoc analysis, while cKO group show similar extent of spending time in all zone($F_{(3)}$=1.07 P=0.37; ANOVA with LST post hoc analysis) *P<0.05 indicates comparision between control and cKO groups, ## P<0.001 indicates target annulus zones vs other annuluses within each group.

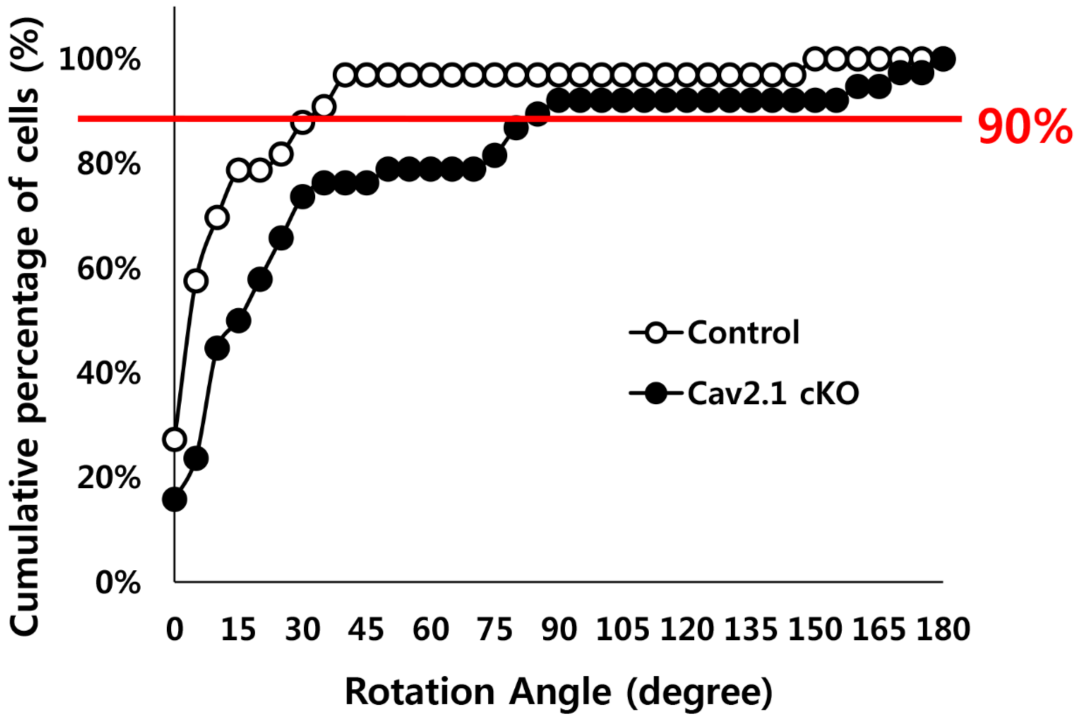

Supplementary Figure S4. Cumulative distribution of rotation angle. Cumulative percentage of place cells at each rotation angle, where each cell has the highest similarity (Max SIM). 90% of place cells in control group have rotation angles within 35 degree. In contrast, 90% in cKO have rotation angles within 90 degree, showing more deviated distribution of angle.

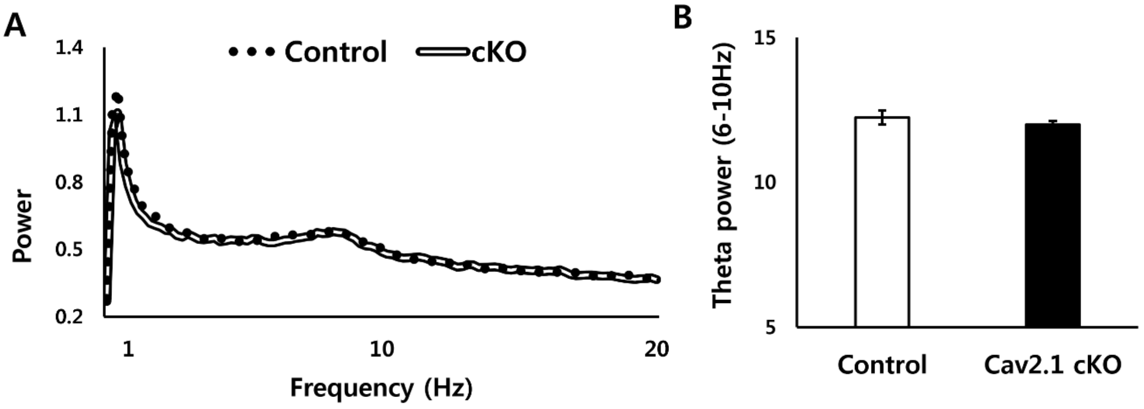

Supplementary Figure S5. Power spectra density of CA1 place cell was analyzed on neuronal spike trains using NeuroExplorer (Nex Technologies). (A) Spectrum of 0-50Hz was obtained and normalized to present power(%) in frequency(Hz) ranging from 0 to 20 Hz. (B) Theta power was calculated within the theta frequency (6-10Hz).

	Control		Cav2.1 cKO	
	Session1	Session2	Session1	Session2
**Firing rate (Hz)**	1.70±0.267	1.92±0.277	1.77±0.231	1.71±0.203
**Mean ISI (ms)**	1.13±0.154	1.13±0.234	1.10±0.167	1.22±0.280
**Field size (cm2)**	162.1±7.31	153.8±6.92	163.3±4.24	162.7±4.33
**In field firing rate (Hz)**	3.09±0.477	3.32±0.449	3.17±0.38	2.95±0.313
**Selectivity**	1.1±0.04	1.0±0.04	1.0±0.03	1.0±0.04
**Coherence**	0.970±0.0021	0.970±0.0023	0.971±0.0025	0.972±0.0019
**Spatial info, bits per s**	3.10±0.203	3.20±0.211	3.09±0.200	2.90±0.185

Supplementary Table S1. Place cell properties between groups during session 1 and 2. All scores were not significantly different between group (two tailed t-test).

	Control	Cav2.1 cKO
**Signed firing rate (Hz)**	-0.052±0.0327	0.001±0.0447
**Signed mean ISI (ms)**	0.052±0.0326	-0.0003±0.0444
**Signed field size (cm2)**	0.025±0.0240	0.002±0.0139
**Signed in field firing rate (Hz)**	-0.039±0.0321	0.011±0.0443
**Signed selectivity**	0.024±0.0129	0.020±0.0134
**Signed coherence**	-0.00014±0.000973	-0.00062±0.001304
**Signed spatial info, bits per s**	-0.021±0.0251	0.034±0.0244
**Unsigned firing rate (Hz)**	0.15±0.022	0.21±0.029
**Unsigned mean ISI (ms)**	0.145±0.220	0.206±0.0286
**Unsigned field size (cm2)**	0.098±0.0172	0.069±0.0080
**Unsigned in field firing rate (Hz)**	0.143±0.0209	0.212±0.0273
**Unsigned selectivity**	0.062±0.0079	0.067±0.0081
**Unsigned coherence**	0.0044±0.00058	0.0056±0.00092
**Unsigned spatial info, bits per s**	0.121±0.0135	0.123±0.0147

Supplementary Table S2. Changes between sessions on individual cell basis. Session1(R1) and session 2(R2) were normalized to observe changes on a cellular basis. Signed value was defined as (R1-R2)/(R1+R2). Unsinged value was the absolute value of the sign. All scores were not significantly different between group (two tailed t-test).
